# Public stigma and treatment preferences for alcohol use disorders

**DOI:** 10.1186/s12913-023-09037-y

**Published:** 2023-01-24

**Authors:** Sara Wallhed Finn, Anna Mejldal, Anette Søgaard Nielsen

**Affiliations:** 1 Unit of Clinical Alcohol Research, Institute of Clinical Research, University of Southern Denmark, J.B. Winsløws Vej 20, Entrance. 220 B, 5000 Odense, Denmark; 2 Department of Global Public Health, Karolinska Institutet, Stockholm, Sweden; 3 Psychiatric Hospital, University Function, Region of Southern Denmark, Odense, Denmark

**Keywords:** Alcohol use disorders; Europe; health care; treatment-seeking; stigma

## Abstract

**Background:**

Alcohol use disorders (AUD) are among the most highly stigmatized medical conditions. Only a minority of individuals with AUD seek treatment, and stigma is one of the most prominent barriers to treatment-seeking. However, there is a lack of knowledge about the associations between stigma and preferences for help-seeking, and the associations between stigma and preferences for treatment seeking.

**Aim:**

to investigate the associations between stigma and preferences for where to seek help and treatment for AUD. As sub-analyses, associations between stigma, level of alcohol use and preferences for help-seeking and treatment preferences will be analyzed.

**Method:**

Cross-sectional design, including *n* = 3037 participants aged 30 – 65 years, living in Denmark. Data: In 2020, an online questionnaire was administered by a market research company. The questionnaire covered demographics, preferences for help-seeking and treatment for AUD, stigma measured with the Difference, Disdain & Blame Scales for Public Stigma, and alcohol use measured with the Alcohol Use Disorder Test (AUDIT). Analyses: restricted cubic spline models were applied to model outcomes. Odds ratios were calculated.

**Results:**

A lower level of stigma was associated with a higher probability of preferring formal and informal help-seeking for AUD. Both high and low levels of stigma were associated with a higher probability of preferring to consult general practitioners. Stigma was not associated with other preferences for treatment-seeking, nor trying to change oneself or a passive strategy. The sub-analyses, grouped by level of alcohol use, showed similar results.

**Conclusion:**

Stigma is associated with lower preferences for formal and informal help-seeking, however not type of treatment preferred. Future studies should address stigma in relation to other factors of the treatment-seeking process.

**Supplementary Information:**

The online version contains supplementary material available at 10.1186/s12913-023-09037-y.

## Background

Public stigma and self-stigma are two different aspects of health-related stigma [1]. Public stigma is defined as negative perceptions and stereotypes, towards a specific group in society, by the majority population [2, 3]. One key aspect of stigma is the perception of differentness, i.e., that the affected group is viewed as different from the majority [4]. This stereotype of differentness is argued as being shared between different stigmatized conditions. Moreover, rather than being just a neutral statement of difference, stigma is considered to involve a perception of differentness that leads to disdain [5]. A person with a medical condition may internalize this public stigma, a process called self-stigma [6]. When stigma is discussed in the present manuscript, however, only public stigma is referred to, i.e., perceptions shared in the general population.

Alcohol use disorders (AUD) are among the most highly stigmatized medical conditions in the Western world [7]. Individuals with AUD are viewed as more responsible for their disorder and elicit more social rejection and negative emotions compared to other disorders. This stigmatization has been found stable over time [8]. Among individuals with substance use disorders (SUD), higher perceived stigma is associated with poorer mental health and lower quality of life [9, 10]. Both the World Health Organization (WHO) and the Substance Abuse and Mental Health Services Administration (SAMHSA) in the United States have emphasized the need for more attention specifically to the stigma associated with addictive disorders [11, 12].

Even though AUD is associated with great harm such as diseases and injuries [13], it has one of the largest gaps between the number of individuals affected and the number of individuals in treatment compared to other psychiatric disorders [14]. Estimates from 26 countries worldwide suggest only 7% of individuals with SUD receive treatment [15]. In Denmark, treatment services for AUD are readily available and free of charge. However, only few individuals seek treatment [16]. In order to reduce the alcohol-related harm, it is important to improve treatment coverage.

It is well established that the stigma associated with AUD, is one of the most prominent barriers to treatment-seeking [17–20]. Previous studies have mainly focused on barriers to treatment and there is therefore a need to also investigate what individuals would potentially do if they developed AUD, i.e. potential preferences for where to seek help, and also potential preferences for treatment seeking, considered by the general population, i.e., not individuals currently in the treatment-seeking process. However, this topic has received little attention, despite calls for more consumer-oriented approaches [21]. Two Swedish studies showed that most individuals would prefer seeking specialist care for AUD within the health care services [19, 22]. Among those suffering from AUD, a preference for seeking treatment in primary care was also stated, as this option was considered less stigmatizing [22]. It is possible that the stigma surrounding AUD is associated with different preferences for where to seek help and treatment. However, so far, there is a lack of knowledge about the associations between stigma and preferences for help-seeking, and the associations between stigma and preferences for treatment seeking in the general population. This study aims to fill these gaps in knowledge, and contribute to the understanding of how to narrow the treatment gap for AUD.

## Aim

The overall aim of this study is to investigate the associations between stigma, preferences for where and how to seek help, and treatment for AUD in the general population.

Specifically, the study will investigate associations between:

1. stigma and preferences for help-seeking;
2. stigma and treatment preferences;

The analyses will take demographic factors, age, sex, education, having children, and previously having alcohol problems into consideration. As sub-analyses, the study will investigate whether associations between

3) stigma and preferences for help-seeking differ by level of alcohol use,
4) stigma and treatment preferences differ by level of alcohol use,

This will be analyzed in a subsample of participants who completed the AUDIT.

## Methods

### Study design

Cross-sectional study

### Participants

The participants were recruited by a market research company with access to a panel consisting of adults from all regions in Denmark. Between June and October 2020, a representative group of adults aged 30 – 65 years was asked to participate in an online questionnaire. The topic of the survey was not known to the participants beforehand. It is not known how large proportion of those asked that opened the survey. The proportion of participants that dropped out before completing the survey was slightly higher compared to similar surveys on other topics: 8.5% compared to normally 5—6%. In total, 3037 individuals participated.

### Outcome

There are two outcome measures: 1) preferences for help-seeking for AUD and 2) treatment preferences for AUD.

Preferences for help-seeking were measured with the question: “What would you do if you developed alcohol problems?”. The participants were presented with the following items, which were examined as separate binary outcomes, and answered yes or no to each: “Try to change it myself”, “Seek professional help”, “Ask those closest to me for help”, “Wait for others to help me”, “Put it off until I found a solution”, or “I would not do anything”. The last three: “Wait for others to help me”, “Put it off until I found a solution”, and “I would not do anything” were grouped together in the analyses and named “Passive strategy”. Treatment preferences were measured with the question: “Who would you contact first if you felt the need to discuss your alcohol use?”. The following alternatives were given, which also were examined as separate binary outcomes, and the participants answered yes or no to each: “General practitioner” (“GP”), “Seek alcohol treatment at the social services”, “Seek advice on the Internet”, and “Call a helpline”.

### Measurements

The online questionnaire covered demographic data on sex, age category, education, and offspring. Experience of AUD was measured with the question:”Have you previously had alcohol problems?”, which could be answered yes or no. Stigma was measured with the Difference, Disdain & Blame Scales for Public Stigma; a questionnaire measuring key aspects of public stigma associated with mental illness. Versions of this questionnaire have been used in previous studies [23, 24]. For the purpose of the present study, the items were rephrased from “mental illness” to “alcohol problems” The questionnaire measures, via three items each, “difference” (example “*How different do you think a person with an alcohol problem is, compared to everyone else in the general population?*”), disdain (example “*How disrespected do you think a person with an alcohol problem is, compared to everyone else in the general population?”*) and blame (example *“How responsible do you think people with an alcohol problem are for their illness?”*). The participants rate a total of nine items on a scale from 1 (Not at all) to 9 (Very much). The score on each item is summed to a total score, the minimum score being 9 and maximum 81, where a higher score indicate a higher level of stigma. Two items were reverse scored before summary. The questionnaire was translated from English to Danish, backtranslated and finalized for the purpose of this study.

The level of alcohol use and related problems were assessed in a subsample of *n* = 1594 participants who also completed the Alcohol Use Disorder Test (AUDIT), a 10-item questionnaire [25]. Please see Appendix 1 for the order of the questions.

### Data analyses

Each item describing preferences for help-seeking and treatment was examined as a separate binary outcome, except for “Passive strategy”, which was a combination of three items. After describing the sample, logistic regression, utilizing restricted cubic splines with three knots, was performed in order to model the associations between the continuous exposure variable, i.e., the total stigma score, and the dichotomous outcomes [26]. This allowed non-linear relationships between stigma and the outcomes. Stigma was modeled with three knots spaced out over the 10^th^, 50^th^, and 90^th^ percentile, corresponding to the following scores: 26, 41 and 52. The reference value was for visualization placed at the stigma value 45, allowing for nine evenly spaced out points on the x axis, with the reference centered. Odds ratios (OR) were calculated with 95% confidence intervals (CI). Cronbachs alpha for the stigma questionnaire was 0.74.

The primary analyses were performed in two steps. First, the crude associations between public stigma and the outcome measures were calculated. Next, all analyses were adjusted for sex, age category, education, having children, and previous experience of AUD. Sub-analyses were performed in the subsample where data on alcohol use and related problems were available (*n* = 1594). These analyses were performed in three steps. First, the sample was divided into two categories; low-risk alcohol use, defined as AUDIT score 0 to 6 for women, and 0 to 8 for men, and hazardous alcohol use, defined as AUDIT score 7 and above for women, and 9 and above for men [25]. Secondly, the crude associations between stigma and the outcome measures were calculated for each category. Thirdly, all analyses were adjusted for sex, age category, education, having children, and previous experience of AUD. All analyses were carried out using Stata MP 16.1 (StataCorp LP, College Station, TX).

## Results

Table 1 presents an overview of the demographics of the participants in the survey and in Appendix 2 the corresponding demographics from the total Danish population [27]. Almost half of the participants were above the age of 50, and a slight majority were females. A third of the participants had children. A majority had more than 12 years of education. 7% of the participants reported previously having problems with their alcohol use. 32% of the participants had an AUDIT score indicating hazardous alcohol use, defined as a score above 6 for women and above 8 for men, and 5% scored above 15 on the AUDIT, indicating a high probability of fulfilling the criteria for alcohol dependence (not shown in the table).

**Table 1:** Demographic

	Total
	*N* = 3037
Sex (female)	1615 (53.2%)
Age category	
30–39	738 (24.3%)
40–49	820 (27.0%)
50–65	1479 (48.7%)
Children	
No	1945 (64.0%)
Yes	1092 (36.0%)
Education	
Up to 12 years	376 (12.4%)
Vocational training	796 (26.2%)
> 12 years	1845 (60.8%)
Missing	20 (0.7%)
Stigma total score	
mean (SD)	39.95 (10.34)
25th percentile	33
75th percentile	46
Previous alcohol problem (yes)	219 (7.39%)
Missing	73 (2.40%)
AUDIT total score	*n* = 1594
mean (SD)	5.98 (4.82)
25th percentile	3
75th percentile	7

**Fig. 1:**
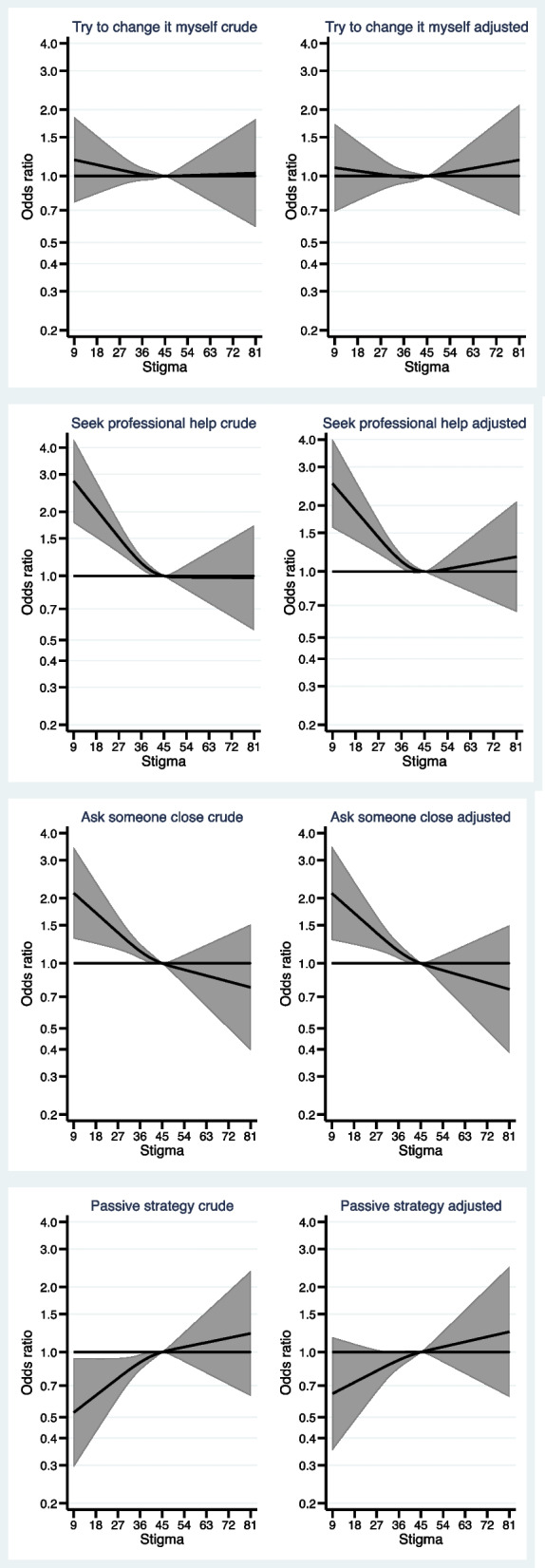
Associations between stigma and preferences for help-seeking *adjusted for sex, age category, education, having children, and previous alcohol problems (*n* = 2650)

Figure 1 shows the association between perceived stigma and the personal preferences for types of help-seeking, when the participants were asked to imagine the need for it. As can be seen from the figure, a lower stigma score, compared to the reference value, was associated with a higher probability of a preference for *seeking professional help* or *ask those closest to me for help*.

**Fig. 2:**
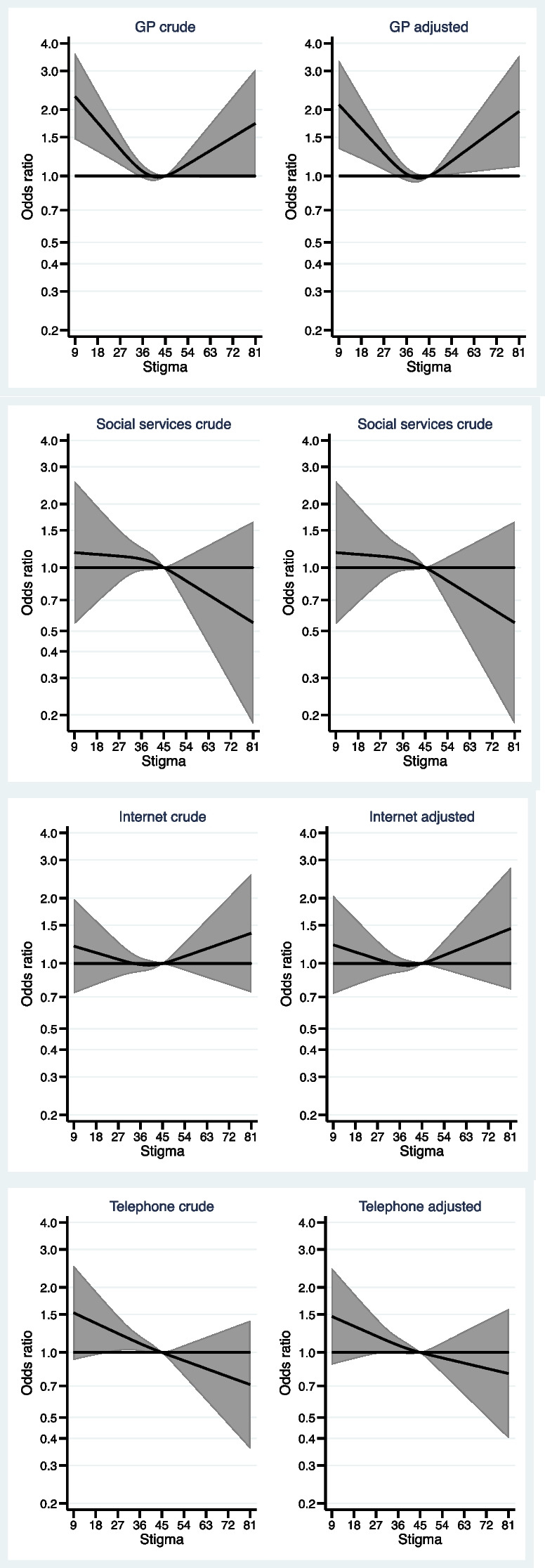
Associations between stigma and treatment preferences Who would you contact first if you felt the need to discuss your alcohol use with someone? *adjusted for sex, age category, education, having children, and previous alcohol problems (*n* = 2650)

Figure 2 shows the association between perceived stigma and preference for type of treatment, when the participants were asked to imagine a need for treatment due to having developed alcohol problems. As can be seen from the figure, both lower and higher stigma scores, compared to the reference value, were associated with a higher probability of a preference for *consulting the GP* in order to receive treatment.

**Fig. 3:**
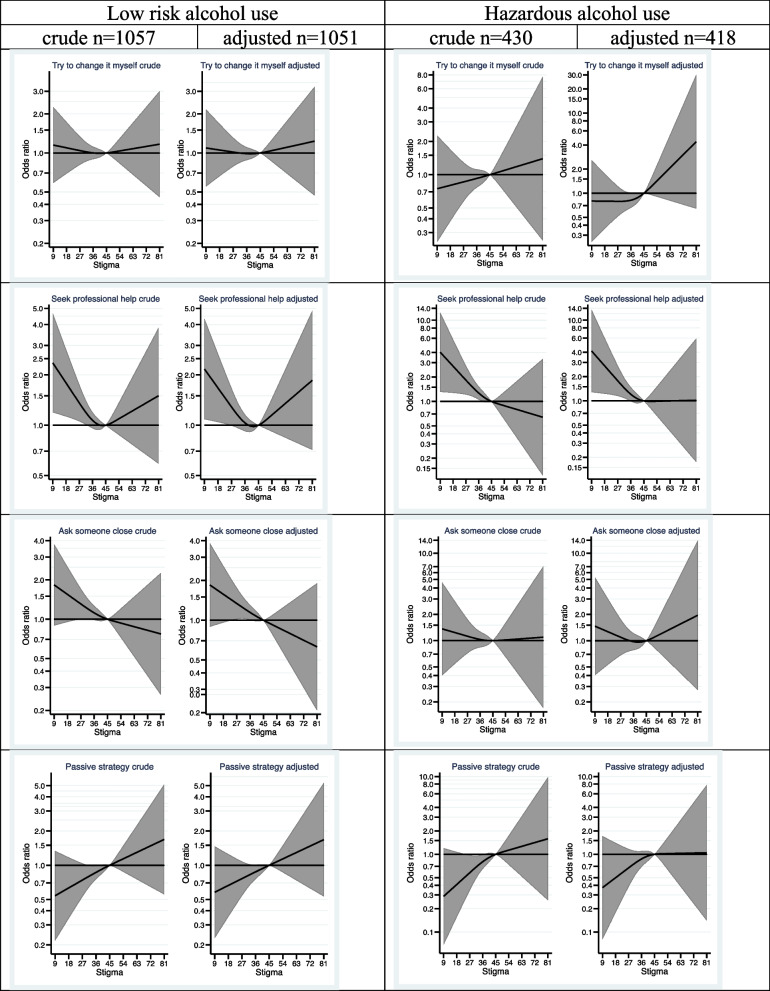
Associations between stigma and preferences for help-seeking, grouped by level of alcohol use *adjusted for sex, age category, education, having children, and previous alcohol problems

Moreover, the association between perceived stigma and preferences for where to seek help when the participants were asked to imagine the need for it, depending on current alcohol use was estimated (Fig. 3). The participants were grouped according to their level of alcohol use: low-risk or hazardous alcohol use, assessed by the total score of AUDIT. The results show that, both among those with low-risk alcohol use and hazardous alcohol use, lower stigma, compared to the reference value, was associated with a higher probability of a preference for seeking *professional help*.

**Fig. 4:**
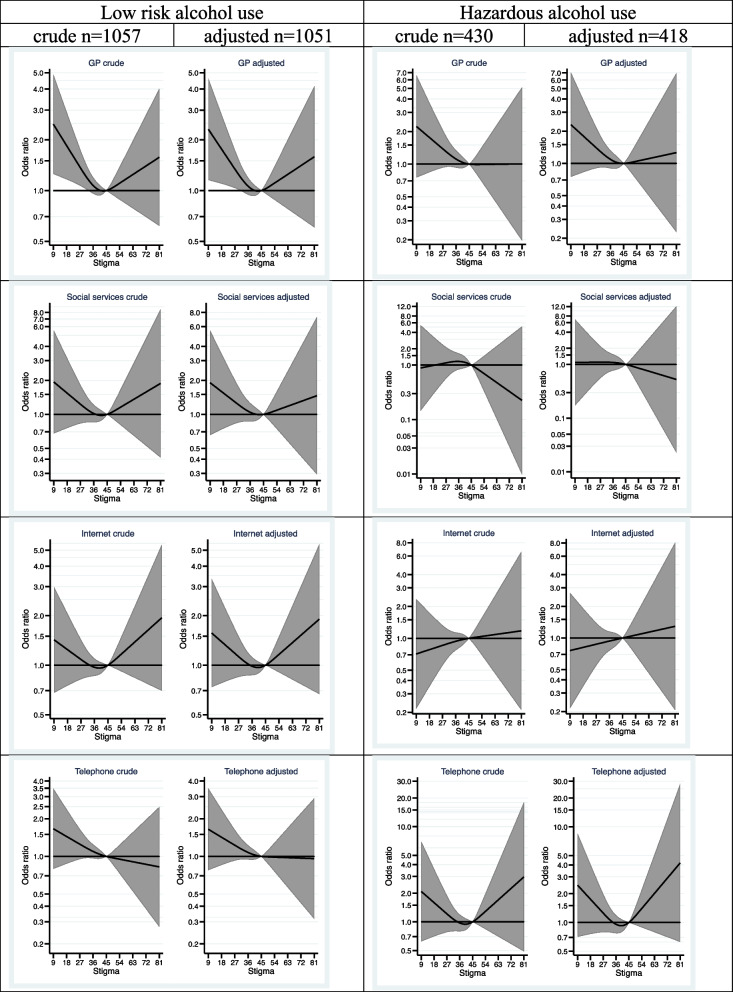
Associations between stigma and treatment preferences, grouped by level of alcohol use *adjusted for sex, age category, education, having children, and previous alcohol problems

Finally, in Fig. 4 the association between perceived stigma and preferences for treatment-seeking, when the participants were asked to imagine the need for it was estimated.

Again, the participants were grouped according to their level of alcohol use: low-risk or hazardous alcohol use. Among individuals with low risk alcohol use, a lower stigma score, compared to the reference value, was associated with a higher probability of a preference for seeking *help from the* GP. This association did not reach statistical significance among individuals with hazardous alcohol use.

## Discussion

The overall aim of the present study was to investigate the associations between stigma, preferences for help-seeking, and preferences for treatment for AUD in a general population sample. Specifically, the study examined associations between: 1) stigma and preferences for type of help-seeking; 2) stigma and preferences for types of treatment; 3) stigma, preferences for types of help-seeking, grouped by level of alcohol use; 4) stigma and preferences for type of treatment, grouped by level of alcohol use. We found that perceived stigma was related to preferences for where to seek help and treatment, and that the individual’s level of alcohol use also had an impact. Our findings will be discussed in detail below.

### Preferences for help-seeking

In our study, based on a survey performed among the general population, a lower perceived stigma was associated with a higher probability of stating a preference for seeking *professional help* and a higher probability of stating a preference for *asking someone close for help*. Sub-analyses, where the participants were grouped by level of alcohol use, still identified an association between lower stigma and a higher probability of a preference for seeking *professional help*, but not an association with a preference for *asking those closest to me for help*. These results indicate that willingness to seek *professional help* is closely related to stigma, and that a high level of stigma may contribute to a reluctance to, and lower preference for, seeking professional treatment. This is in line with the research on barriers to treatment, which shows that the stigma associated with AUD is an important barrier to treatment-seeking [17–19]. Thus, in order to reduce the current treatment gap, there is an urgent need to reduce the public stigma attached to AUD. Educational-based interventions and social contact interventions have shown to be effective in the short term, both for reducing stigma associated with mental health in general and SUD in particular [28, 29]. However, there is a need for high qualitative research in this field and studies on effects over a long-term follow up. The use of continuum beliefs for messages around treatment-seeking for AUD (e.g. framing AUD as part of a continuum rather than a dichotomous disorder) could be especially relevant for future studies, as it has also been found to reduce stigma for psychiatric disorders [30]. There is also evidence that messaging, applying a continuum beliefs model of AUD compared to a binary belief model, can increase problem-recognition, which in turn can improve treatment-seeking [31].

Stigma may have different mechanisms and impose different types of barriers to help-seeking depending on the type of help – e.g., seeking professional treatment or informal support from the social network. Regarding stigma and its association with seeking informal support, it is known that health-related stigma in general, but not specifically for AUD, is associated with not telling others and fear of social rejection and judgment [32]. Moreover, the present study also showed that public stigma in the general population decreases the preference for informal support-seeking for AUD, when the issue is considered hypothetically. Among individuals in treatment for SUD, higher stigma is found to be associated with lower perceived social support [9, 33]. Similar findings are made among individuals living with HIV [34]. It is, however, unknown to what extent these findings illustrate a reciprocal process with decreased social support, or rather withdrawal from social support, possibly because of fear of social rejection.

A preference for avoiding help or treatment from the outside, and rather trying to change on one’s own, was not associated with the level of stigma nor seemingly affected by one’s own alcohol use, which may not be surprising. Epidemiological studies show that many with AUD recover without seeking help [35], although treatment-seeking has been found to increase the rates of recovery [36, 37]. The wish to handle alcohol use on one’s own, has also been reported as a reason for not seeking treatment for AUD [18, 38]. The present study suggests that there are mechanisms other than stigma, associated with this preference. A qualitative Swedish study showed that a contributing factor to the wish to handle one’s alcohol use on one’s own was the perception that AUD was a bad habit, which could be altered by changes in everyday life, rather than seeking treatment [18]. This indicates that the personal framing and understanding of AUD contributes to preferences on how to solve it. This is also in line with other studies, showing that the choices of treatments strongly relate to the perceptions of the causes of AUD [39, 40].

### Treatment preferences

Regarding preferences for where to seek treatment, the results of the present study showed that both lower and higher stigma scores were associated with a higher probability of a preference for consulting a GP for treatment. In the sub-analyses, when alcohol use was also adjusted for, the association between lower stigma and a higher probability of a preference for consulting a GP was also found. These findings emphasize the crucial role that GP’s and primary care play as a cornerstone in Danish health care [41]. It also suggests that the trust in GPs is high, and that GPs are expected to play an important role in addressing and treating AUD. Other studies have also found strong support for routinely asking questions about alcohol use in primary care [42]. Thus, GPs play a vital part in recovery from AUD [43, 44].

There were no associations between stigma and preferences for seeking treatment through social services, the Internet and by telephone, even when the level of alcohol use was taken into consideration. This is surprising, considering that a previous study on treatment preferences showed that Internet support and telephone helplines were preferred alternatives for assessment and guidance to treatment [18]. Moreover, it is often possible to access Internet support or telephone helplines anonymously, which has been reported to lower the threshold for seeking support for stigmatized health conditions as AUD [45, 46]. A possible explanation for this finding may be that the treatment-seeking process—in itself – is associated with stigma. Non-treatment-seeking adults with AUD have reported that the need for treatment is, in itself, shameful and a sign of failure [18, 45]. It has been found that seeking treatment is associated with a perceived change of identity toward a stigmatized stereotype of someone with AUD [18, 45]. Similar observations have been made among individuals seeking treatment for SUD [47]. This could be seen as an example of a self-stigma process, and the results from the current study suggest that, in relation to stigma, the choice of *where* to seek treatment is secondary to the decision *to* seek treatment. Future studies should investigate this further.

### Strengths and limitations

An important limitation is the use of self-report measures on topics such as stigma and alcohol-related questions, which can be perceived as sensitive by the participants. Sensitive topics pose an increased risk for giving socially desirable answers. In order to reduce the risk of bias, stigma was measured with a questionnaire that emphasizes differentness, which is considered to impose less risk for biased answers compared to other measures of stigma [48, 49]. However, we acknowledge a lack of psychometrically sound and brief instruments to measure public stigma in general, and public stigma associated with AUD specifically. This is a potential threat to the validity of the measure of stigma in this study, and an important limitation.

Another limitation is that the current study only measured preferences for different treatment settings. It is possible that factors other than the setting are important, such availability, or messages about treatment goals – reduced alcohol use, or abstention only.

AUD, stigma and help-seeking are complex phenomena, where synergistic effects between these and factors such as socioeconomic position, gender, and age are to be expected. Moreover, previous studies have shown that individuals familiar with AUD, and individuals with lived experience of AUD, are less likely to endorse stigmatizing attitudes towards others with AUD [50]. A strength of the current study is that these factors were included, together with a large sample size. A limitation is the age range among the participants, between 30 and 65 years, which hampers the possibilities to generalize the findings to other groups. Another is the lack of information about the proportion of invited participants who answered the survey.

## Conclusion

Stigma is associated with a lower probability of a preference for seeking both professional help and informal help-seeking. Both lower and higher stigma was associated with a higher probability of a preference for consulting a GP, emphasizing the important role of primary care in addressing and treating AUD. However, stigma was not associated with other treatment preferences. Future studies should address stigma in relation to other factors of the treatment-seeking process for AUD, such as the understanding of AUD, the decision to seek treatment, and specific messaging characteristics around the treatment settings.

## Supplementary Information

**Additional file 1**.

**Additional file 2**.

## Data Availability

The data underlying this article cannot be shared publicly due to the privacy of individuals that participated in the study. The data will be shared on reasonable request to the corresponding author.
