# Supplementary material for: Public stigma and treatment preferences for alcohol use disorders
Source: BMC Health Serv Res. 2023 Jan 24;23:76. doi: 10.1186/s12913-023-09037-y (PMC9872434; doi:10.1186/s12913-023-09037-y)
Supplement: Supplementary file 1 — Additional file 1. [file 12913_2023_9037_MOESM1_ESM.docx]

Appendix 1

Order of the questions

1. Demographic data
2. AUDIT
3. Previous experience of own AUD
4. Preferences for help seeking

*Items presented in a randomized order*

1. Preferences for treatment

*Items presented in a randomized order*

1. Difference, Disdain & Blame Scales for Public Stigma
