# Supplementary material for: Public stigma and treatment preferences for alcohol use disorders
Source: BMC Health Serv Res. 2023 Jan 24;23:76. doi: 10.1186/s12913-023-09037-y (PMC9872434; doi:10.1186/s12913-023-09037-y)
Supplement: Supplementary file 2 — Additional file 2. [file 12913_2023_9037_MOESM2_ESM.docx]

Appendix 2

Demographic

|  | Total | Total  Danish population  Year 2020 |
| --- | --- | --- |
|  | N=3037 | Age 30-65 years  N= 2 652 880 |
| Sex (female) | 1615 (53.2%) | 1 320 600 (49.8%) |
|  |  |  |
| Age category |  |  |
| 30-39 | 738 (24.3%) | 686 808 (25.9%) |
| 40-49 | 820 (27.0%) | 756 048 (28.5%) |
| 50-65 | 1479 (48.7%) | 1 210 024 (45.6%) |
|  |  |  |
| Education |  | Age 30-64 years  N= 2 590 614 |
| Up to 12 years | 376 (12.4%) | 587 210 (22.7%) |
| Vocational training | 796 (26.2%) | 904 931 (34.9%) |
| >12 years | 1845 (60.8%) | 1 058 401 (40.9%) |
| Missing | 20 (0.7%) | 40 072 (1.5%) |
